# *Nothing really changed*: Arizona patient experience of methadone and buprenorphine access during COVID

**DOI:** 10.1371/journal.pone.0274094

**Published:** 2022-10-25

**Authors:** Beth E. Meyerson, Keith G. Bentele, Danielle M. Russell, Benjamin R. Brady, Missy Downer, Roberto C. Garcia, Irene Garnett, Rebecca Lutz, Arlene Mahoney, Savannah Samorano, Christina Arredondo, Honey J. Andres, Haley Coles, Brenda Granillo

**Affiliations:** 1 Southwest Institute for Research on Women, College of Social and Behavioral Sciences University of Arizona, Tucson, Arizona, United States of America; 2 Family and Community Medicine, College of Medicine, University of Arizona, Tucson, Arizona, United States of America; 3 School of Social Transformation, Arizona State University, Tempe, Arizona, United States of America; 4 Center for Rural Health, Mel and Enid Zuckerman College of Public Health, University of Arizona, Tucson, Arizona, United States of America; 5 Comprehensive Pain and Addiction Center, University of Arizona, Tucson, Arizona, United States of America; 6 Southwest Recovery Alliance, Phoenix, Arizona, United States of America; 7 Drug Policy Research and Advocacy Board, Tucson, Arizona, United States of America; 8 Sonoran Prevention Works, Phoenix, Arizona, United States of America; 9 El Rio Health, Tucson, Arizona, United States of America; University of New Mexico Health Sciences Center, UNITED STATES

## Abstract

**Objective:**

To understand patient experience of federal regulatory changes governing methadone and buprenorphine (MOUD) access in Arizona during the COVID-19 pandemic.

**Methods:**

This community-based participatory and action research study involved one-hour, audio-recorded field interviews conducted with 131 people who used methadone and/or buprenorphine to address opioid use disorder at some point during COVID (January 1, 2020- March 31, 2021) in Arizona. Transcribed data were analyzed using *a priori* codes focused on federally recommended flexibilities governing MOUD access. Data were quantitated to investigate associations with COVID risk and services access.

**Results:**

Telehealth was reported by 71.0% of participants, but the majority were required to come to the clinic to attend video appointments with an offsite provider. Risk for severe COVID outcomes was reported by 40.5% of the sample. Thirty-eight percent of the sample and 39.7% of methadone patients were required to be at the clinic daily to get medication and 47.6% were at high risk for COVID severe outcomes. About half (54.2%) of methadone patients indicated that some form of multi-day take home dosing was offered at their clinic, and 45.8% were offered an extra day or two of multi-day doses; but no participants received the federally allowed 14- or 28-day methadone take-home doses for unstable and stable patients respectively. All participants expressed that daily clinic visits interrupted their work and home lives and desired more take-home dosing and home delivery options.

**Conclusions:**

MOUD patients in Arizona were not offered many of the federally allowed flexibilities for access that were designed to reduce their need to be at the clinic. To understand the impact of these recommended treatment changes in Arizona, and other states where they were not well implemented, federal and state regulators must mandate these changes and support MOUD providers to implement them.

## Introduction

At the outset of the U.S. COVID pandemic, people taking medication for opioid use disorder (MOUD) were caught in a catch-22: visit a methadone clinic or buprenorphine provider and risk COVID exposure and serious health outcomes, or avoid the clinic and risk treatment failure or overdose [1]. The pandemic had a deleterious impact on people who use drugs (PWUD) because of reductions in treatment [2] and harm reduction-related services [3]. A 17.6% increase in U.S. overdoses [4] was initially reported as a consequence of the COVID-19 crisis. Currently, U.S. overdose deaths increased 32.0% since 2019 [5].

Even before COVID, MOUD access was inequitably distributed by geography [6, 7], payment [8], and race/ethnicity [9, 10]. Treatments are regulated differently as well. Methadone treatment in the United States requires daily dispensing and supervised consumption visits at licensed opioid treatment programs (OTPs or methadone clinics), and limited “take-home,” multi-day doses [11]. Buprenorphine, conversely, is prescribed by licensed providers with pharmacy dispensing following an in-person medical evaluation [12]. These disparate treatment structures are due to regulations embodying the racialization of methadone and the carceral approach to the use of illicit substances and to substance use disorder in the United States generally [13, 14]. As such, MOUD patients are essentially a class experiencing disparate treatment access and outcomes due in large part to this regulatory bifurcation.

In March 2020, federal regulators enacted several temporary policy changes to enhance MOUD access and reduce COVID exposure in response to the emerging pandemic [15–17]. These changes allowed telehealth services including treatment induction, multi-day take home doses of up to 14 and 28 days for unstable and stable patients respectively, alternate delivery of medications, reduced drug testing and diversion callbacks.

Notably, these changes were not mandatory, so it is likely that institutional, organizational, and provider-level factors impacted MOUD access during COVID. The true effects of these policy changes are slowly becoming apparent. Current published research suggests a continuum of policy implementation by MOUD providers [18–20]. A study conducted in Texas and New Mexico among methadone treatment providers found varying perceptions about the benefits of multi-day take home dosing and concerns about provider liability and patient safety, including some who opposed them as threats to patient-centered care [21]. A Washington state OTP case study described provider concerns about blanket access to multi-day take home doses [13]. Even with potential benefits from regulatory flexibility, an Arizona study raised concerns that telehealth might exacerbate patient isolation, may alienate people without technological access, and likely replicates the Foucauldian medical gaze making patient circumstances even more apparent to providers [22]. These concerns counterbalance a different, persistent access issue in Arizona that might be solved by telehealth: distance [23].

Whether to continue or even expand these regulatory flexibilities remains a question for research consideration because there is bias against MOUD based on myths about people who use drugs, about those who are addicted to substances, and myths about treatment misuse and diversion [24].

To understand the impact of MOUD policy flexibilities on patient safety and access to care, it is also important to understand patient perspectives. Little is known about MOUD treatment systems or access to them in Arizona or much of the Southwest United States and especially during COVID. The purpose of this study is to characterize Arizona patients’ policy experience. We examined how patients experienced federal regulatory changes while accessing MOUD during COVID.

## Methods

This study has a community-based participatory and action research (CBPAR) orientation [25]. An Arizona-wide, transdisciplinary group of people with lived (illicit drug use) experience, harm reduction partners, MOUD providers, MOUD patients, and academic researchers was formed to develop the study goals, methods, guide the implementation of the study, make sense of the findings, and plan for dissemination. This group is called the DPRAB: Drug Policy Research & Advocacy Board.

Data were collected through 60-minute, in-person interviews with people who were at least 18 years of age, living in Arizona, and MOUD patients at some point during COVID (defined as January 1, 2020-March 31, 2021). Interviews were conducted between August-October 2021. A 27-question instrument in English and Spanish measured aspects of MOUD access experience during COVID, experience of federal regulatory flexibilities, perceptions about their MOUD experiences, recommendations for MOUD access improvement, and health risks associated with COVID in the past 2 years.

To measure COVID risks, we asked about health conditions over the past two years identified by the U.S. Centers for Disease Control and Prevention (CDC) as being associated with severe COVID outcomes (hospitalization and death) [26]. These included: Immunocompromising conditions such as HIV, cancer treatment or organ transplant; chronic kidney disease or undergoing dialysis; chronic lung disease or moderate to severe asthma; diabetes; liver disease; and serious heart condition. We also asked about other health issues common to people who use drugs such as hepatitis C, tuberculosis, skin and soft tissue infections, endocarditis, chronic wounds, abscesses or cellulitis, and diagnoses of anxiety, depression or schizophrenia.

Interviews were conducted by individuals with lived illicit drug use experience. This approach facilitated trust and comfort in sharing about stigmatized and sometimes illegal behavior [27]. Interviewers were hired, trained and financially supported to recruit and interview participants for the study. As this was a qualitative study, generalizability was not possible; however, interviewers represented the diversity of Arizona, including bilingual (Spanish/English) language and geographic location (county and rurality). Interviewers also had experiences with cross-sections of communities such as those who are undocumented, rural and BIPOC communities, and linkages to both treatment modalities of methadone and buprenorphine. Interview recruitment was social and through convenience sampling conducted by interviewers themselves. Given the diversity of Arizona, planned recruitment was to be large (above 100) to assure theoretical saturation.

As this study took place during COVID, interviewers were trained on zoom, and conducted in person interviews using an audio recorder allowing safe social distancing while preserving privacy. Personal protective equipment was provided for interviewers and participants. Interview participants were offered a $30 visa gift card for participation. Participants were provided study information in writing and verbally for an informed consent process. This was an oral process whereby the study information sheet would be retained by the participant and they would give verbal assent on the recorder to proceed with participation. Once a participant gave verbal agreement to the interviewer, they would be asked again for consent once the audio recorder was turned on and if agreeing to participate, stated “I agree to participate in this interview.” This agreement became part of the transcribed audio recording for a record of consent once the interview was transcribed.

Interviewers were paid for training time, to conduct the interviews and upload them to a HIPAA compliant portal for transcription. They also convened with co-authors (BEM, DMR, MD) to make sense of the initial data and observations in preparation for the DPRAB review of data. Audio recordings were anonymous, and uploaded interviews were deleted from recording devices within 12 hours of upload and completeness confirmation. The University of Arizona institutional review board of the Human Subjects Protection Program provided ethical oversight for this study.

Interview data were transcribed and coded using *a priori* categories focused on evidence of federal regulatory flexibility, experiences with MOUD access during COVID, and risk of severe COVID outcomes. For coding purposes, there were three groups of patients: methadone only, buprenorphine only, and those accessing both independently during COVID. Qualitative data were quantitated to allow description and associations testing with outcome variables of COVID risk and reported regulatory flexibilities using Pearson chi-square goodness of fit, Fisher’s exact test and t-tests as appropriate. Statistical significance was noted at the p ≤ .05 level. Qualitative data are reported where appropriate to provide context and rich description. Quantitative and qualitative data were managed and analyzed using SPSS (IBM, version 28) and QSR NVIVO (QSR International, version 1.6.2) respectively.

## Results

The sample included 131 people. Rural communities were overrepresented as compared with Arizona population distributions (16.8% in sample vs. 4.7% in Arizona) [28], as were people in Pima County (home to Tucson) (35.8% of sample population vs 15.1% in Arizona), and smaller communities of Mohave (6.8% of sample vs. 3.1% in Arizona), Yuma (13.0% of sample vs. 3.1% in Arizona), and Yavapai (6.8% of sample vs 3.4% in Arizona). Maricopa, home to Phoenix and surrounding suburbs of Mesa and Tempe, represented 37.4% of the sample population as compared with being 66.2% of the state’s population [29]. In terms of racial and ethnic diversity, 32.1% of the sample self-identified as BIPOC (Black, Indigenous and people of color) which included all participants who self-identified as one or more of the following: Hispanic, Black, Asian, Indigenous or multi-racial. In 2020, BIPOC individuals represented 17.4% of Arizona’s overall population [30].

The sample overrepresented the distribution of buprenorphine patients among MOUD patients (25.2% in sample vs. 13.2% in 2015 nationally; Arizona data not available) [31]. Participants received treatment from 29 different providers of methadone and/or buprenorphine across the state and reported a mean time on buprenorphine of 35.5 months, whereas people on methadone reported a mean treatment time of 37.6 months. Among all people on MOUD, including buprenorphine, roughly half (51.9%) had to go to the clinic daily to receive their medication; with a mean distance of 4.8 miles from the provider and mean one-way travel time of 23.4 minutes. See Table 1.

*Just getting down there was a challenge*. *I did not have a driver’s license…*..*so*, *having to coordinate rides through medical transportation should be seamless*. *Unfortunately*..*there just always seemed like there was some sort of complication*, *they would show up in the wrong address*, *they would show up late*, *show up early*. *And so it would wind up sometimes I wouldn’t actually get picked up until hours later*. *And so what should just have been a 30-minute*, *40-minutes maybe trip down there…*. *wound up being an entire day process of scheduling medical transportation*, *making sure that I’m there during a certain window*, *coordinating with dispatch to make sure that the driver gets to me to pick me up from my home…*..*Five hours give or take from start to finish*. (Participant 66, Mesa)

**Table 1:** Characteristics of people on MOUD during COVID, Arizona (N = 131).

Demographics	
Age	Mean = 37.9 yrs (r:19–67, SD: 11.1)
Race and Ethnicity	
White	89 (67.9%)
Hispanic	31 (23.7%)
Black	4 (3.1%)
Native American	4 (3.1%)
Asian	3 (2.3%)
Gender Identity	
Cismale	78 (70.9%)
Cisfemale	50 (38.2%)
Nonbinary	3 (2.3%)
Sexual Orientation	
Heterosexual	111 (84.7%)
Bisexual	14 (10.7%)
Lesbian or Gay	4 (3.1%)
Queer	2 (1.5%)
**Housing**	
Housing was affected by COVID	57 (43.3%)
Unhoused at some point during COVID	20 (15.3%)
Rurality (Towns listed in population order by rurality)*	
**Rural**	22(16.8%)
Cordes Lakes (2,684 pop)	1 (0.8%)
Dewey-Humbolt (4,326 pop)	1 (0.8%)
Sommerton (14,197 pop)	3 (2.3%)
Kingman (32,689 pop)	9 (6.9%)
San Luis (35,257 pop)	1 (0.8%)
Prescott (45,827 pop)	7 (5.3%)
**Rural/Urban Mix**	15 (11.5%)
Tempe (180,587 pop)	2 (1.5%)
Yuma (203,881 pop)	13 (9.9%)
**Urban**	94 (71.8%)
Scottsdale (241,361 pop)	1 (0.8%)
Mesa (504,258 pop)	16 (12.2%)
Tucson (542,629 pop)	47 (35.9%)
Phoenix (1,608,139 pop)	30 (22.9%)
**MOUD Access During COVID**	
Methadone only	96 (73.3%)
Buprenorphine only	33 (25.2%)
Both Methadone and Buprenorphine	2 (1.5%)
Had to go to the clinic daily to get medication	68 (51.9%)
Total time on methadone (in months), predates COVID	Mean = 37.6 (r:1–240, SD:41.1)
Total time on buprenorphine (in months), predates COVID	Mean = 35.5 (r:3–73, SD:18.1)
**Distance from MOUD Provider**	
Miles from provider	Mean = 4.8 (r:1–25, SD:4.4)
Commute time to provider (in minutes *one way*)	Mean = 23.24 (r:5–90, SD:15.7)

*U.S. Decennial Census 2020 (www.census.gov)

### Risk for severe outcomes from COVID among people on MOUD

Under half (40.5%) of the sample reported at least one underlying condition placing them at high risk for severe COVID outcomes, as identified by CDC. If these conditions were combined with mental health diagnoses which are also known indicators of COVID risk [32], 68.7% of the sample was at risk for severe COVID outcomes. For the purpose of this paper, risk of severe COVID outcomes is measured as having at least one of the underlying conditions identified by CDC. See Table 2.

**Table 2:** Risk of severe COVID outcomes among people on MOUD in Arizona during COVID by selected covariates, 2021 (N = 131).

	Total# (% total sample), N = 131	High Risk for COVID Severe Outcomes per CDC (n = 53)
		#(% subsample)
Methadone only patient	96 (73.3%)	42 (43.8%)
Buprenorphine only patient	33 (25.2%)	9 (27.3%)
Both Methadone and Buprenorphine	2 (1.5%)	2 (100%)
Urban	94 (71.8%)	40 (42.6%)
Rural	22 (16.7%)	9 (40.9%)
Urban/Rural mix	15 (11.5%)	4 (26.7%)
Unhoused at some point during COVID	20 (15.3%)	9 (45.0%)
Time on Buprenorphine (Months)	Mean = 35.5	Mean = 33.0 months
Time on Methadone (Months)	Mean = 38.6	Mean = 49.2 months*
Age (years)	Mean age = 40	Mean = 40.4 yrs*
BIPOC	42 (32.1%)	15(35.7%)
Had to go to the clinic daily to get medication (all MOUD)	68 (51.9%)	26 (38.2%)
Had to go to the clinic daily (Methadone only)	52 (39.7%)	20 (47.6%)

*Independent samples t-test yielded mean differences at the p ≤ .05 level.

Nearly half of methadone patients were at high risk for COVID (43.8%), and their time on methadone was a mean of 49.2 months as compared with those on methadone who were not at high risk (38.6 months). Of those on methadone treatment, 39.7% had to go to the clinic daily, and 47.6% of this group reported being at high risk for severe COVID outcomes. Far fewer buprenorphine patients were at risk for COVID (27.3%), but some still had to go daily to their clinic or provider to receive medication. As would be expected, risk for COVID severe outcomes increased with age. As time on MOUD increased, particularly for those on MOUD between 31–60 months, the risk for COVID severe outcomes increased. Notably, 38.2% of all people required to be at the clinic daily to get medication were at high risk for COVID severe outcomes.

*Yeah*, *it didn’t matter*. *No changes* (during COVID). *You had to go every day until you were clean for a while*, *yeah*. *Still have to do it…*.*They spread the chairs out*, *but they definitely weren’t six feet*, *you know*? (Participant 115, Kingman)

*Yeah*, *that was a daily thing*. *And then it was a lot of drug tests*, *especially I think because I was pregnant*, *and then the whole COVID thing…*. *I would also have to wait outside the clinic and wait for them to drug test me*. (Participant 80, Prescott)

### Federal regulatory flexibilities experienced by patients

For participants who were on MOUD prior to the pandemic, few changes to their MOUD access were reported during COVID. Most reported the same types of requirements as prior to the pandemic: urine testing, daily clinic visits for methadone, evidence of no opiates in system for up to 72 hours (buprenorphine), intensive outpatient classes with cancellations as the only change, and meetings with a case manager or counselor.

*It’s about the same as before*, *honestly*. *It wasn’t a huge change in terms of some of the things*. (Participant 23, Tucson)

Table 3 displays the federal regulatory flexibilities experienced as service changes by people on methadone and buprenorphine during COVID. Among all federal flexibilities, a majority (71.0%) of participants experienced telehealth services at some point during COVID. Living in an urban area was significantly associated with receiving telehealth services. Participants appreciated telehealth especially when it reduced their having to be at the clinic. But for many participants, telehealth services were not structured to keep them at home because they were required to come to the clinic where they used a clinic computer or phone to speak to a provider who was offsite.

*You would go in for your appointment and you go into a small room and be on video screen*. *You would just see the provider that way*, *through the computer*, *like a Zoom type of thing*. (Participant 72, Tucson)

*The truth is that I’m not in favor of video calls or video-calling; I don’t like that*. *I’d like to have somebody here* (at the methadone clinic) *in person that could hear me*. *I don’t feel safe in a video call*, *and that’s what most agencies offer here*, *just video calls*. (Participant 60, Yuma)

*I guess the insurance wouldn’t pay for phone to phone audio*, (Medicaid) *wouldn’t pay for it*. *So now you have to go down to the office and sit and do audio and video*, *but* (the provider) *is still working from home because insurance*… *I don’t know*. *I guess it was getting expensive*. (Participant 129, Prescott)

**Table 3:** Counts and proportions of federal regulatory flexibilities offered to MOUD patients during COVID, Arizona 2021 (N = 129)^^^.

Federal Regulation Flexibility	Total (% total sample, N = 131)	Methadone Only #(% of n = 96)	Buprenorphine Only #(% of n = 33)	Mean Time on MOUD (months)	High risk for COVID per CDC #(% of n = 53)	BIPOC #(% of n = 42)	Rural #(% of n = 22)	Urban #(% of n = 94)	Rural- Urban Mix #(% 0f n = 15)
Telehealth (offered or required)	93 (71.0%)	67 (69.8%)	24 (72.7%)	M = 36.2	36 (67.9%)	32 (76.2%)	19 (86.4%)	61 (64.9%)*	13 (86.7%)
Multiday take-homes offered at clinic	73 (55.7%)	52 (54.2%)	n/a	M = 29.6	32 (60.4%)	28 (66.7%)	14 (63.6%)	53 (56.4%)	6 (40.0%)
Multiday take-homes offered to participant	63 (48.1%)	44 (45.8%)	n/a	M = 28.4	27 (50.9%)	27 (64.3%)**	10(45.5%)	47 (50%)	6 (40.0%)
Curbside dosing offered	38(29.0%)	35 (36.5%)**	n/a	M = 44.2	17 (32.1%)	9 (21.4%)	7 (31.8%)	30 (31.9%)	1 (6.7%)
Alternate delivery offered	20 (15.3%)	10 (10.4%)**	9 (27.3%)*	M = 29.6	8 (15.1%)	6 (14.3%)	2 (9.1%)	17 (18.1%)	1 (6.7%)
Reduced drug testing	24 (18.3%)	16 (16.7%)	8 (24.2%)	M = 30.9	8 (15.1%)	9 (21.4%)	4 (18.2%)	17 (18.1%)	3 (20.0%)
Reduced diversion callbacks	17 (13.0%)	13 (13.5%)	4 (12.1%)	M = 23.7	6 (11.3%)	7 (16.7%)	5 (22.7%)	8 (8.5%)	4 (26.7%)

*Association significant at the p < .05 level

**p ≤ .01 level using either Chi Square with Pearson goodness of fit or independent samples t-test.

^The two patients who were on methadone and buprenorphine during COVID were not included in this analysis due to cell size.

Just over half of participants (55.7%) attended clinics where multi-day take home doses were offered to patients, and 48.1% reported receiving any type of multi-day take home dose. This means that 86.3% of the people who were at clinics that offered multi-day take home doses were offered them at some point during COVID. This number was slightly smaller for methadone patients. While everyone offered additional take-home doses appreciated them and wanted more of them, among all MOUD patients, *no one* reported receiving the maximum allowed for stable patients (28 days) or the maximum allowed for unstable patients (14 days). In fact, most of the stable patients (urinalyses negative for other drugs) reported receiving far fewer than the possible 28 day take-home dosing. Notably, the longer people were on MOUD (mean of 43.4 months vs. 29.6 months), the less likely they were to report that multi-day take home doses were offered at their clinic, and they were less likely to have multi-day doses offered to them personally (mean of 42.1 months vs. 28.4 months).

When asked what they would like to see to improve their treatment access, every participant asked for more multi-day take home doses and for home delivery. It was this flexibility that was the central change needed to allow patients to live a full life with work and family responsibilities without being tied to the clinic.

*If they could bring it right to my door before I leave*, *so I don’t have to go anywhere but to work*, *that would be awesome*. (Participant 111, Tempe)

(It) *was a pain in the ass because the closest* (clinic) is in Bullhead. *So they got to pick you up at five o’clock in the morning*, *drive you down there in the bus…But they pick you up at this time*, *and you got to be there at this time*. *And then even to see their doctors*, *you have to go all the way down there to see the doctor* (45 minutes). *And there’s no guarantee you’re going to get your dose that day*. *And you have to sit there and wait and you make the bus wait*. *Well*, *after picking everybody up*, *you’re looking at like two hours*, *something like that…*.*That’s why I stopped going to them because I had to go to work*. *And there was no way I could make it all the way there to talk to the doctor and get everything set up*, *and then make it to work on time*. *There’s no possible way*. (Participant 115, Kingman)

Curbside dosing for methadone, while helpful to reduce COVID exposure, was offered to 29.0% of patients–mostly urban. Out of the 6 people voluntarily reporting being positive for COVID in the last 18 months (at the time of interviews), 4 were offered curbside dosing. Alternative delivery of medications to reduce COVID risk was also largely not offered to methadone patients (90.0% did not receive this). Among the 10 people on methadone who received this flexibility, they expressed appreciation and felt supported.

*Oh man*, *they were shut down for a while*. *You either got on where they bring it to you*, *which the Tribe is really good*, *they don’t let their people suffer*, *so they were bringing it to us…*.*The nurses would come to you and give you your dailies; sometimes they couldn’t make it because there’s so many of us*. *So if you’re on Methadone and you don’t get your fix*, *you’re sick*. *What are you going to do*? *You’re going to relapse*. *No matter what*. *You’re going to start shaking*, *sweating*, *shitting on yourself*, *and you’re going to go get some heroin*, *or you’re going to go get some pills*. (Participant 47, Tucson)

Finally, 12.2% (n = 16) of participants voluntarily indicated (no standard question was asked) that the clinic stopped offering any of the flexible services identified, even as they themselves were never offered them. This was reported by participants throughout the interview period (August-October 2021). Of those who observed the rescinding of services, 14 were methadone patients. The changes were primarily the removal of telehealth services and any multi-day take home doses, if offered.

*During the time that I started on MAT services* (October 2020), *they were back to normal*. *I heard that they were doing it a little bit different*, *but I didn’t get to experience that part*. (Participant 37, Phoenix)

*They stopped at my clinic probably like six months ago*, *eight months ago* **(November 2020-January 2021)**, *they stopped doing take homes***. (Participant 52, Phoenix)**

## Discussion

Assessing the impact of sweeping regulatory changes to MOUD treatment during a pandemic is daunting. Evidence is emerging about implementation, views about the federal policy recommendations, and experiences of them throughout the country [33, 34]. However, a full picture of peer-reviewed evidence has yet to emerge. Many published studies occurring during 2020 were on the east or west coast of the U.S. and were small in size.

To our knowledge, this study is among a very few to report direct patient experience with policy changes in MOUD access during COVID. A telehealth satisfaction survey was conducted in one Rhode Island provider setting [35], a survey study of North Carolina patients in three methadone clinics was reported [36], and a very small, non-peer reviewed study in one San Francisco methadone clinic with 10 providers and 20 patients reported views about methadone take-home dosing [37]. The North Carolina study found that take-home doses increased from pre-COVID periods to the time of the study (summer 2020) from 56–82% to 78–100%, though notably, the clinic-level percent of patients receiving a week or longer (>6 days) ranged from 11–56%. In contrast, our study recruited 131 people on MOUD across Arizona and involved experiences from numerous and different providers, treatment modalities and settings.

Our findings contrast with more than a few provider-based studies in which providers reported implementing federal policies to enhance MOUD access during COVID. Trietler et al’s study among a small group of New Jersey MOUD providers reported a shift towards telehealth, a reduction in toxicology screening and counseling services, and modifications to multi-day dosing [38]. A study of a San Francisco methadone treatment program reported full implementation of federal regulations (largely undefined) with outcomes demonstrating the benefit of increased take-home doses on hospitalizations and emergency department visits [39]. The closest alignment of provider reporting and patient experience is a multi-state study by Krawczyk of unpublished data reported to HHS Office of The Inspector General from methadone clinics. Krawczyk’s findings were similar to ours. Their data suggest that while a majority (89%) of methadone clinics increased take home doses, only 26.8% of patients received a 14-day take home dose and 16.8% received the 28-day take home dose [40].

The discrepancy between what patients and providers report indicates a need for additional, comparative studies with larger samples *in the same locations* to assure confirmation of what is really happening in a jurisdiction. The current literature also highlights the need for more nuanced language. There is a different finding when asking ‘did you increase take-homes’ as compared with asking ‘how did you increase take homes’, ‘for what percentage of patients’, and ‘for how long?’ Reported studies suggest difficulties with systems change and practice change. Without knowing provider opinions we can only surmise why such changes were not offered to even stable patients on methadone. Perhaps, as was suggested, providers need much more clarity about characterizing patient stability [40].

Arizona is one location where federal MOUD access flexibilities were generally not experienced by patients. This may be a function of MOUD provider implementation challenges, provider beliefs about various flexibilities, or a function of the significant leeway states have with implementing policy changes about substance use treatment [41]. Arizona’s Medicaid program essentially echoed federally allowed flexibilities but did not require them. However, the legislature mandated that all health plans provide coverage for services through telehealth at the same level as in-person services of the same type [42]. This is likely because patients across health conditions needed it, and not because of the needs of people on MOUD. But as noted by at least one participant, telehealth was not necessarily funded by insurance, including the state’s Medicaid program, and many patients were required to come to the clinic to use telehealth. In this case, the benefit was to the provider and not the patient in terms of COVID risk reduction.

There are a few limitations to this study. One was the fact that we could not explore experiences of buprenorphine patients who indicated (n = 16) they were required to go to the clinic daily for medication. A second limitation was that we did not directly ask participants whether the clinic was still offering whatever flexibility they experienced. What we reported here is a voluntary disclosure and not representative of the entire sample. Third, even as a qualitative study, we could not verify the representativeness of this study population with the population of Arizona MOUD patients because such data are not available. Finally, these data reported here are entirely from patient self-report. The study did not verify access by review of patient health records.

During COVID, there were no regulatory barriers to improving access, and the evidence does not support that relaxing them was problematic. What remains a problem is that providers and organizations are not voluntarily adopting practices that will benefit MOUD patients. Thus, perhaps mandating the federal policy changes would allow patients to fully access MOUD and for us to truly study the impact of these changes on such highly regulated treatments. In the absence of full regulatory implementation, we will not understand what normalizing MOUD treatment on par with other chronic health treatments would look like in the United States. If we never improve MOUD treatment access flexibility, people on MOUD will continue to face deadly consequences due to lack of access, and policy making partners will continue to blame the patient for the range of system-created deleterious health outcomes.
